# Etiology of Dental Caries

**Published:** 1883-01

**Authors:** 


Etiology of Dental Caries.—It is an encouraging sign when so many of the best men in dentistry are turning their attention toward the study of the etiology of dental caries. Most practitioners have heretofore been content to consider the best means of limiting the progress of this disease, without consideration of its origin, or making any effort toward bringing about a change in the condition which resulted in tooth decay. A few thinking men have experimented with widely varying results. In England and in Germany various theories have been propounded, mostly founded upon the hypothesis of a chemical solution of the inorganic portions of tooth structure. In this country, that positive contradiction of the *mens sana in corpore sano*, that sound mind in an unsound body, George Watt, long ago, to his own satisfaction at least, distinctly traced the various kinds of tooth decay to distinctly separate acids, but despite all this the question is yet a vexed one. More recently, such men as Boedecker, Atkinson and Abbott, have pushed the study of histology into regions previously unexplored, but the results so far reached are not yet quite satisfactory. Dr. W. D. Miller, an American dentist residing in Berlin, Germany, has made, and is now making exhaustive researches into the pathology of caries, but the results which he has reached are not entirely in harmony with those of most other investigators, though some of his deductions are unquestionable. The latest writer upon this subject is Dr. C. T. Stockwell, of Springfield, Mass., whose observations and experiments lead him to the conclusion that chemistry has very little, if anything, to do with the matter, but that bacteria are, first and last, the active agents which induce caries. Thus it may be seen that in this country alone there is a wide diversity of opinion, of which George Watt upon the one hand, and Dr. Stockwell and Prof. Mayr upon the other, are the extremes, and the problem is as far from a satisfactory solution as ever. But each intelligent investigator is letting in a little more light, and we may hope that the day is not far distant when enough testimony will have been adduced to warrant the formulating of a theory which shall be consistent with all the known facts.

Two years ago, the American Dental Association, recognizing the importance of the subject, formed a new section entirely devoted to the consideration of physiology and etiology, and began to encourage investigation in this direction. Previous to this time, so far as our knowledge goes, etiology had seldom been discussed in that body. At the last meeting another step was taken, and a prize of two hundred dollars was offered for the best paper upon that subject, to be based upon original investigations. It is to be hoped that the results of this offer may be something worthy the subject. Yet even now so few of the members of that body are interested that but three men chose to be enrolled in that section. The opportunities for unlimited talk on the basis of a very slender amount of original observation are so much greater in other sections, that it is little wonder that the speech-makers seek those fields. But there is already a promise of better things, and next year it is to be hoped that the consideration of the causes of the decay of teeth will occupy much of the attention of our more important dental societies. The INDEPENDENT PRACTITIONER purposes to have something to say upon this subject during the coming year.

